# Understanding the biochemistry of hormones – message in a bottle

**DOI:** 10.1042/EBC20240039

**Published:** 2025-02-06

**Authors:** Dominic C. Y. Lai, Jonathan Wolf Mueller

**Affiliations:** Department of Metabolism and Systems Science, College of Medicine and Health, University of Birmingham, Birmingham, UK

**Keywords:** difficult-to-teach subject, anthropomorphic storytelling, anthropomorphism, innovative teaching material

## Abstract

Hormones play pivotal roles in our well-being, and even more so in times of stress or disease. They determine body composition and govern reproductive processes. Hormonal compounds tend to be evolutionarily very old compounds, but only coevolved receptor systems make up powerful biological signals. We will discuss what makes some metabolites good building materials for hormones and how information may be encoded, using these scaffolds. Starting with hormone biosynthesis and regulated release from secreting cells, we will look at different stages of the whole hormone signaling process: the distribution of the hormonal “message-in-a-bottle” throughout the body, the passing of some hormones through membranes, and pre-receptor metabolism. Binding to different classes of receptors is not the end of hormone signaling, but the beginning of a second phase of signaling via second messengers, before hormonal messages are switched off again. Studying hormone biochemistry will produce exciting new findings in the future.

## What is the purpose of this article?

A lot of biomolecules are dealt with in A-level and undergraduate teaching. Many of these might be signaling compounds, even more so if we also look at plant hormones and bacterial signaling molecules. Only some of them might be connected to the signaling of hormones in the human body. Hormones are not always in their native state; they need to be chemically synthesized, modified, secreted, transported, taken up again, and maybe modified again before they might bind to their cognate receptor and elicit a biological response. All this happens according to the laws of distribution in an undirected manner. Never does a hormone migrate exactly to where it is “needed”; NEVER. We then apply a lot of these aspects to the family of steroid hormones. Chapters on the analytics and research around hormones, disorders of hormone excess and defect, as well as the evolution of hormonal signaling systems may be insightful for the interested reader. The present article concludes by widening the concept of what a hormone might be; it actually is anything that the organism is able to make and to detect again so that a specific biological response is triggered. Hormonal biochemistry may bring promises and surprises in the future.

## Introduction

**Figure 1:**
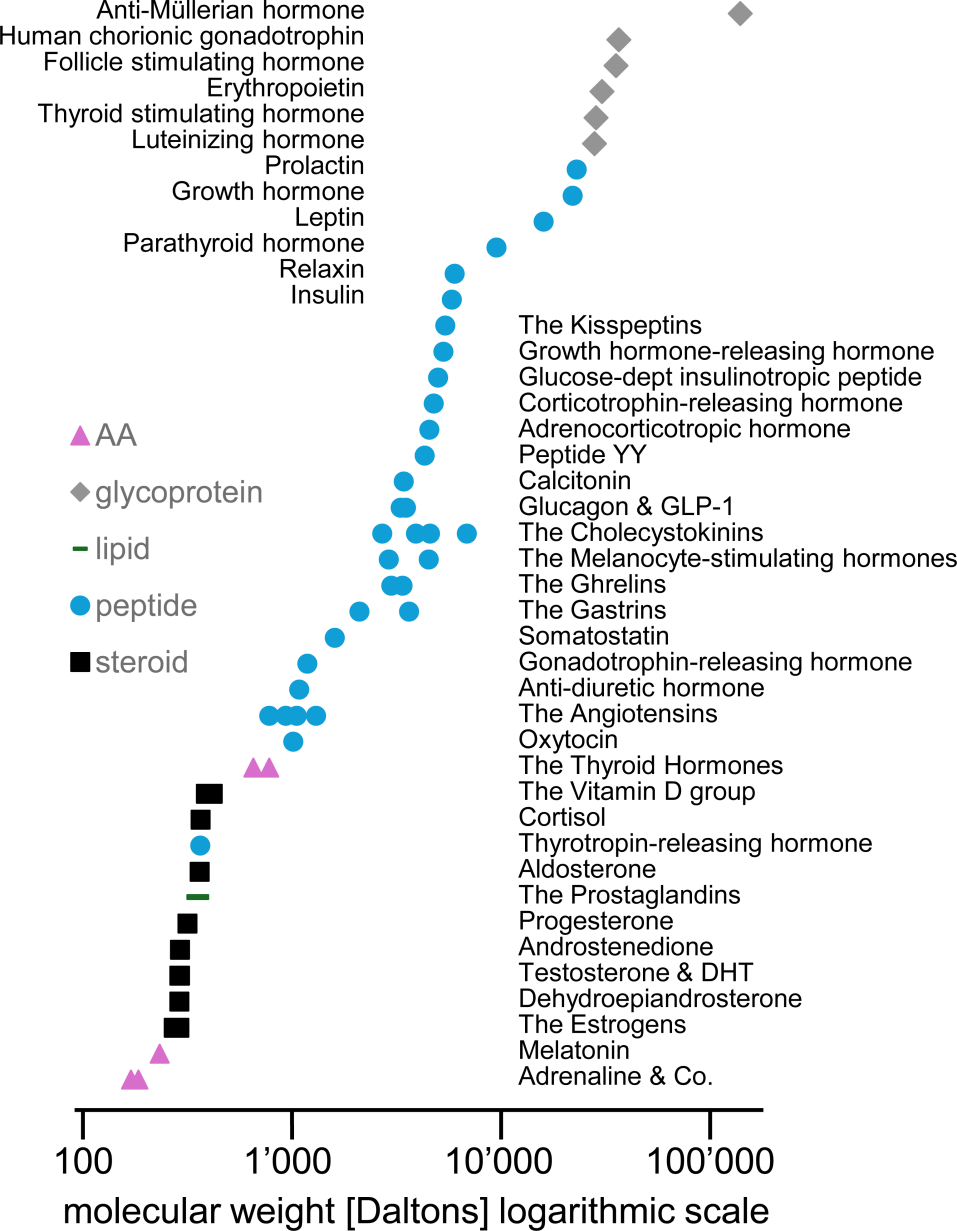
Hormones are chemically diverse. Human hormones or hormone families are listed, together with an approximate molecular weight and an indication of the compound class. This list has been taken from the UK Society for Endocrinology’s educational website www.yourhormones.info, with minor changes in the grouping of hormones, resulting in 42 different hormones or families of hormones. Yes, 42 again. Fun fact: While adrenaline and noradrenaline feature amongst the smallest hormones, the anti-Müllerian hormone seemingly is a hormonal signaling giant. Should one of the authors of this review take this personal?

This article is from the *Understanding Biochemistry* series, and it focusses on hormones – those powerful master regulators of all sorts of human bodily processes that are important for healthy homeostasis and even more important in challenging times. Figure 1 shows the **hormones, or groups of hormones**, that the UK Society for Endocrinology lists on their educational webpage www.yourhormones.info, all regulating various aspects of human physiology. We will discuss aspects of hormonal biochemistry, drawing from this rich collection, limiting ourselves, however, to some illustrative examples only, instead of covering complete hormonal systems.

What are “our” hormones then, the human hormones? A cell may be challenged by environmental circumstances; a cell may need certain nutrients. It is then that this cell may send out a chemical message to get help. Chemical communication between cells is the norm, not the exception; **hormones** are those chemical signals that **act body-wide**, also known as systemic. For hormones, the sensor cell, the cell sending the hormonal message, and the responding cell may easily be three different cells found in different tissues or even organs, far apart within our body.

Moreover, that list of hormones is not necessarily complete; meaning, even if we stick to human physiology only, it is by no means certain that we already know all of the human hormones. Then, there are many other important **signaling molecules** that are not included in this list which strictly might not be regarded as hormones – neuronal ones – neurotransmitters and neurohormones, local signals for signaling between neighboring cells, lots of intracellular signaling, and more. Other animals, as well as plants, fungi, and bacteria, might use vastly different molecules for their chemical communication. Hence, that list is quite an anthropocentric view of what hormones are, as we started off focusing on the human body only.

**Figure 2:**
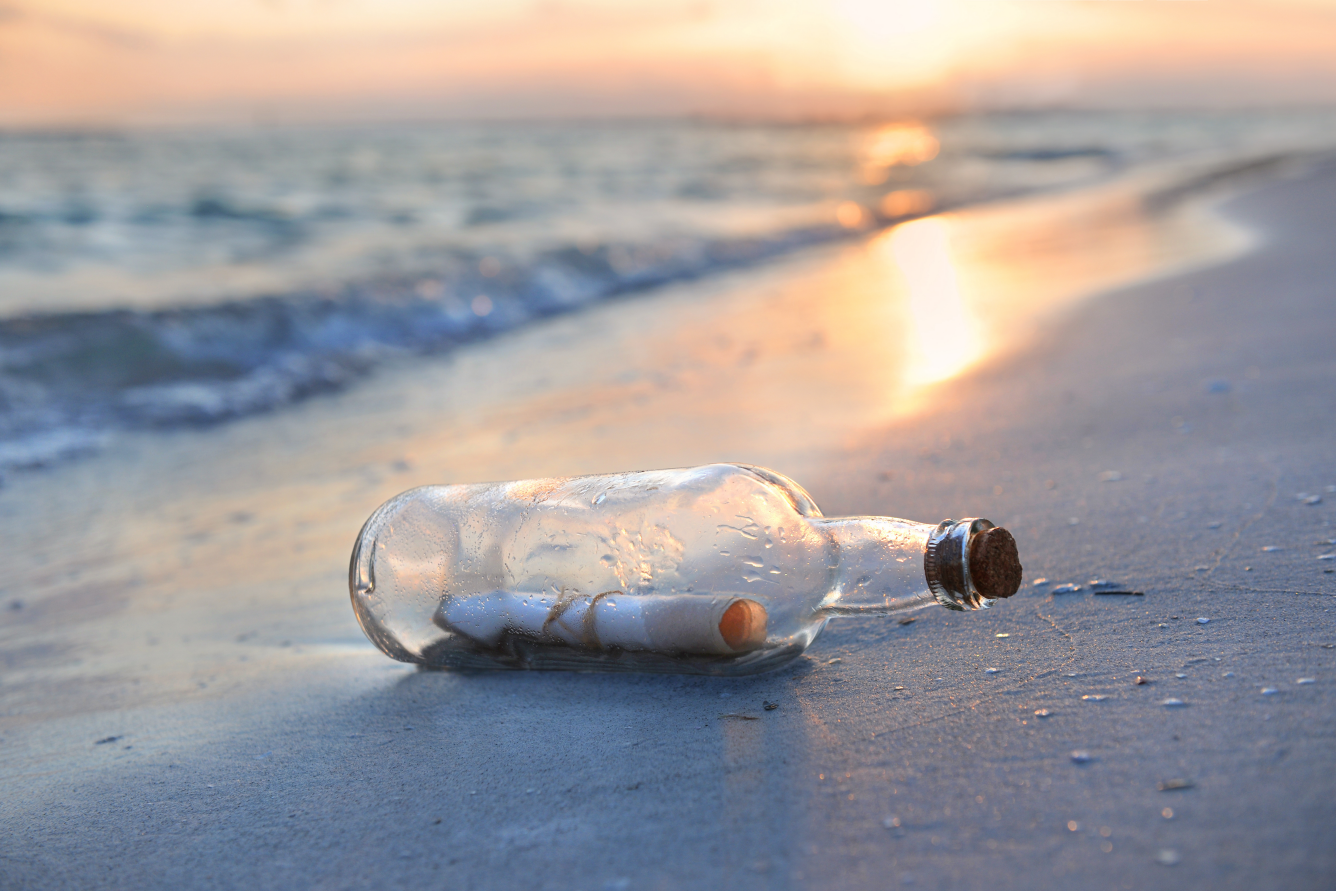
If you send out a hormonal message, make sure you send it in sufficient quantities. A message-in-a-bottle thrown into an ocean equates to ONE particle within that vast volume of liquid. How would it be if some gland in our body secreted ONE molecule of a hormone, just one copy of the hormonal message to be sent out? Let us assume that one hormone molecule would travel through all circulating blood, but it would not be able to distribute to other bodily parts. Some chemical calculations later, and thinking of Avogadro’s number, this one particle dissolved in 5 l, makes up a tiny molar concentration of around 0.35 × 10^−24^ moles per liter. This is less than one particle per liter. Should that one hormone find its receptor at all, it could only stimulate a single cell in the body, not causing any significant signaling at all. Cortisol is a special substance, our fight-or-flight hormone, supposed to make us fit for work. Daily cortisol levels reach a maximum in the morning. In the UK, the average 8–10 am morning cortisol level in blood is 0.35 micromoles per liter or higher. How much more is this concentration compared to the hypothetical molar concentration of our message-in-the-bottle? Handily, the two “0.35” cancel each other out. So, this comparison is all about setting the concentration from above, 10^-24^ [for fans: a yocto-mole] and the cortisol levels in micromole per liter, 10^-6^, into perspective. Astonishingly, the concentration of cortisol would be 10^18^ higher than the concentration of that ONE message-in-the-bottle in our circulation; that is a million • a million • a million times more. Such a massive fold increase in concentration would certainly cause an effect compared with that lonely molecule. Image credit: R. Gino Santa Maria via AdobeStock.

We will explore how and where hormonal compounds are made biochemically. Primarily, these are enzyme-catalyzed reactions, but sometimes rather special chemistry is at work. We will discuss when hormones are secreted into bodily fluids. Hormonal compounds diffuse through our body, passively. Very much like a message-in-a-bottle, a hormone molecule cannot “choose” where to go (Figure 2). There is **a numbers game around hormones**, always searching for the optimal amount of a given hormone. How much is enough? How much is too much, or too little, and could it give rise to disease? In Figure 2, we try to calculate a theoretical molar concentration of one “molecule” message-in-a-bottle; then these numbers are related to the concentration of our stress hormone cortisol in our body – just before we wake up – the morning cortisol level.

At certain places in our body, however, some mechanisms are in place that make that random distribution of hormonal messages seem like a targeted shipping exercise: This can be shortcuts in circulations, dedicated portal vein systems, specific transporters, or elaborate uptake systems. Maybe confounded by these exceptions, our first- and second-year students tend to have difficulties with the concept of **randomness of distribution** of hormonal ‘messages’, and drug-like molecules alike.

For this article, a patchwork of thoughts and anecdotes around hormonal biochemistry emerges that hopefully helps to understand key concepts in this exciting field – please think of that message-in-a-bottle again, at times…

## How old are hormones?

**Figure 3:**
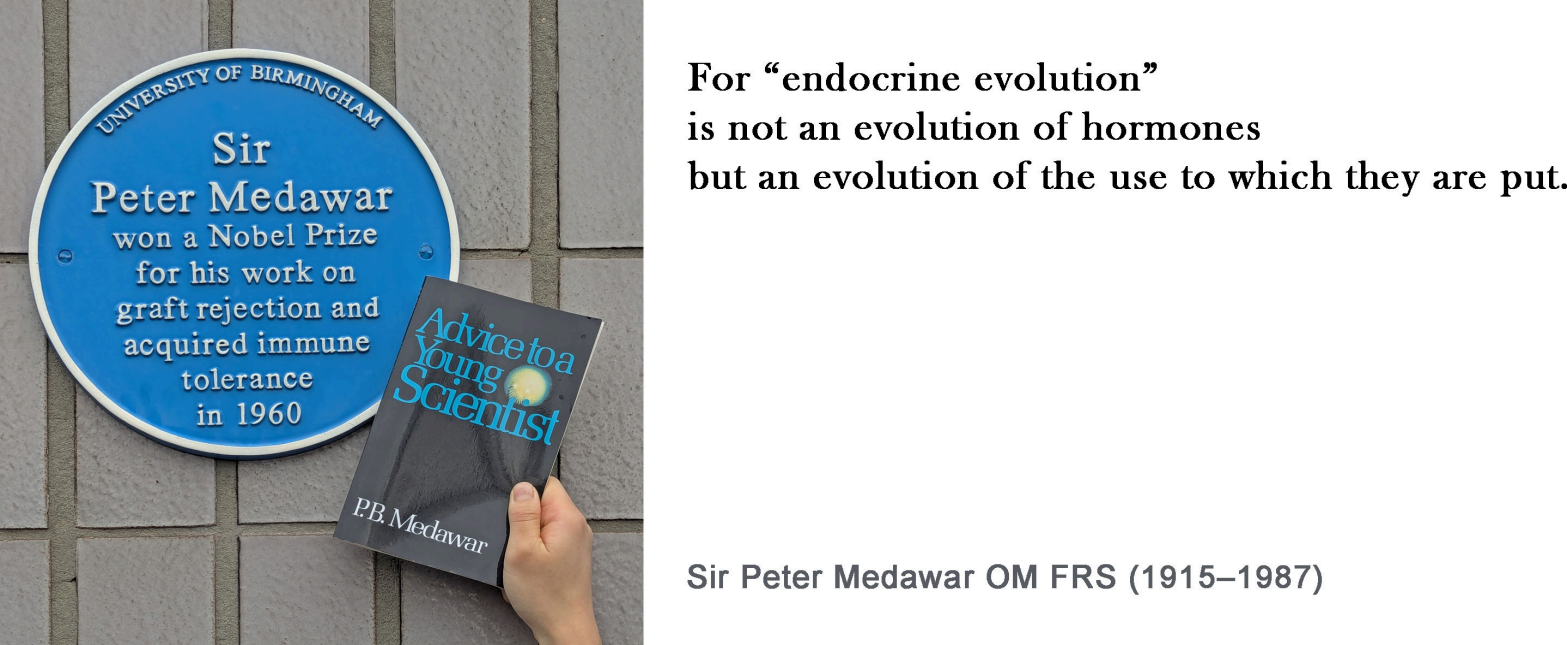
Peter Medawar’s endocrine dictum. “For ‘endocrine evolution’ is not an evolution of hormones but an evolution of the use to which they are put.” A lot has been written by Peter Medawar and about him. He made major contributions about graft rejection and the discovery of acquired immune tolerance. A great number of quotations are attributed to him, amongt them the dictum here presented. Peter Medawar more or less invented the genre of scientific counseling literature and founded the staff bar in the now-gone National Institute of Medical Research in Mill Hill, London.... and he was Professor of Zoology at the University of Birmingham, that is why the authors of this article photographed his Blue Plaque on our campus, on a blurry winterly day.

The molecules we call hormones today tend to have a long **evolutionary past**, as well as an extended history of their scientific discovery. Being around for billions of years seems to be a good predisposition for a substance to become a hormone. Even though we will discuss some hormones from an evolutionary angle further down in this paragraph, this assumption is not correct. A quote by Peter Medawar most famously highlights the issue (Figure 3).

Applied to **steroid hormones**, for example, the specific hormonal compound, such as testosterone, might have been around for long; what evolved, however, might have been the uses it has been put to. This may involve the shaping of the receptor system, the modifying enzymes, the uptake and secretion transporters, and other components to interpret the same chemical message, such as testosterone, into different contexts.

We arrived at **comparative endocrinology**. Within the field of steroids, some astounding differences between humans and mice might illustrate my point – *Of Mice and Men*: When mice get stressed, they do not secrete cortisol, but another glucocorticoid, corticosterone. The human tandem of cytochrome P450 enzymes CYP11 B1 and B2, which produce mineralocorticoids and glucocorticoids, respectively, is not shared by rodents and a lot of other mammals either. Irrespective of the above, mice do not have a *zona reticularis* in their adrenal glands. Taken together, steroid hormones might be evolutionarily older than some surprisingly recent ways of using them in hormonal communication.

**Figure 4:**
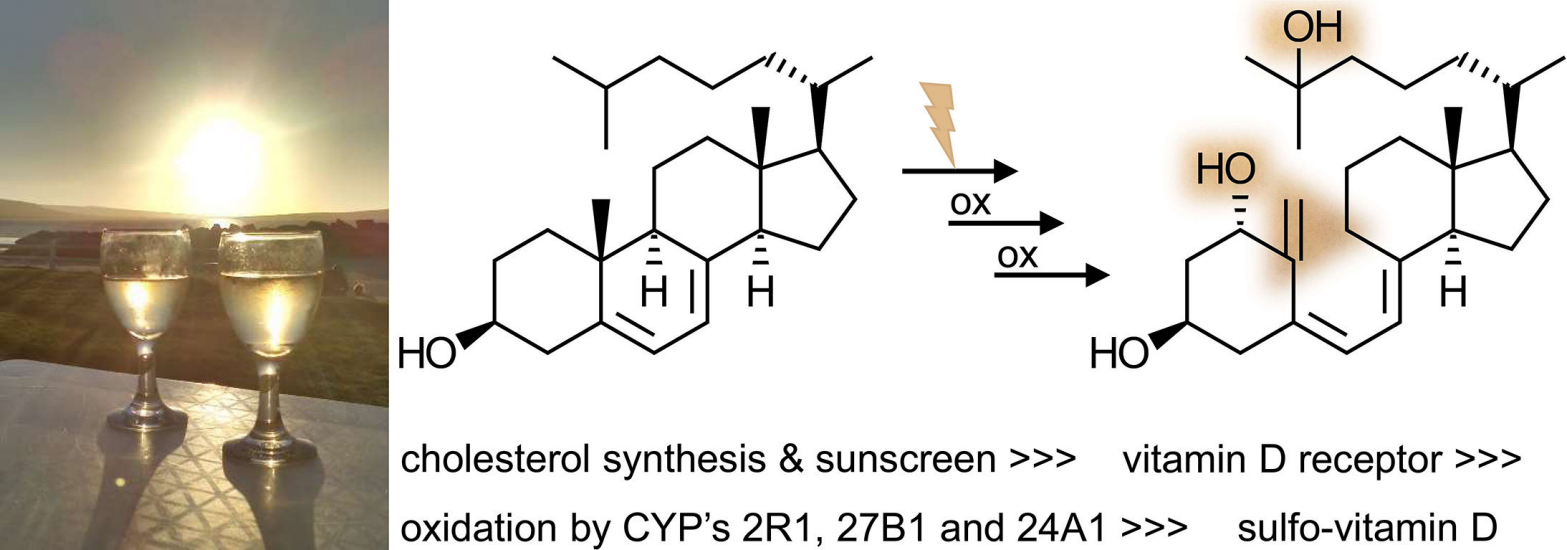
A narrated timeline for the evolution of the vitamin D3 signaling system. For our ’sunshine hormone’ to regulate all the processes, it regulates these days, several molecular entities had to evolve. This is a ‘narrated’ timeline only, because it might be difficult to establish an exact order of events. Cholesterol biosynthesis is believed to be 1200 million years old. 7-Dehydrocholesterol, as an intermediate of today’s cholesterol biosynthesis must have been around equally long. Chemically, that ‘middle’ ring of 7-dehydrocholesterol, the one with some double bonds, is something special. It is not an aromatic compound. That bond can only be cleaved photochemically. Hence, the first use of 7-dehydrocholesterol was as sunscreen during sun exposure. Even today, photosynthetic phytoplankton accumulates this compound on sunny days. Maybe 550 million years ago, the vitamin D receptor evolved from other nuclear receptor genes. A number of oxidation steps then had to shape the ligand for this receptor; only the doubly hydroxylated 1,25-hydroxy-vitamin-D3 happened to be the high-affinity signaling compound. Meanwhile, we know that there are several transport events around the uptake and distribution of vitamin D3 in our body, some of them include the conjugation of a sulfate moiety. In humans, vitamin D3 regulates calcium and bone metabolism; impacting also on the innate immune system and adaptive immunity. Vitamin D3 signaling has become so important that humans have adapted to it, first by depositing 7-dehydrocholesterol specifically in the skin, and later by establishing lighter skin pigmentation in areas with lower levels of sunlight. Image credit: Jon Wolf Mueller

A second example shines light on endocrinology. About a billion years ago, there were already some forms of life that accumulated the pre-vitamin D3 molecule 7-dehydrocholesterol in their cells, when the sun was shining upon them. 7-Dehydrocholesterol, also known as pre-vitamin D3, would absorb UV light and rearrange double bonds to form **vitamin D3**, among other byproducts, depicted in Figure 4. This UV light-absorbing system would protect other parts of the cell from UV-induced damage. What started off as a molecular UV sunscreen only later became an endocrine system around vitamin D, our sunshine hormone. For this to happen, an intricate enzymatic system for activating vitamin D and a specific nuclear receptor had to evolve. More recently, the addition of sulfate completed this system.

Insulin as well as insulin-related signaling peptides, are all tiny proteins; some would even call them just peptides. Apparently, insulin-related signaling is very old, as even the roundworm *Caenorhabditis elegans* has an insulin receptor gene. Medawar’s dictum can nicely be applied here, as the worm does not care about its blood glucose levels ‘yet’ – it does not even have blood. For the worm, insulin and up to 40 related insulin-like peptides mainly serve as anabolic signals for growth and differentiation. Mutations in this signaling pathway drastically change the lifespan of worms.

A hormone that has only made it recently onto the above list of hormones is the signaling peptide **kisspeptin**. This is not because it evolved lately, but because it was discovered only in the 1990s in the lovely town of Hershey, Pennsylvania, famous for candy, well, for Hershey’s kisses. This is what kisspeptin has its name from. Only some years later, kisspeptin was identified as the natural ligand of the G-protein receptor GPR54, an orphan receptor until then. GPR54 was found to be expressed in the human brain, pituitary, and spinal cord. It turned out to be the master regulator of gonadotropin-releasing hormone-secreting neurons, and thus the initiator of puberty and sexual development. What a pun, ‘it all starts with a kiss’, with the kisspeptin gene *KISS1*.

Nevertheless, we know that there are at least two answers to the question about the age of a specific hormone. One is the age of the signaling molecule itself; the other is about the signaling system around it. It seems that a ‘good’ signaling molecule, maybe even stemming from the prebiotic era, has the tendency to stick around, while its signaling context is steadily modulated. Then, there is the **question about complexity**: Have hormone signaling systems become more or less complicated through evolution? A response in reflex would be that signaling certainly only could have gotten more complicated. However, taking the insane insulin signaling of worms as an example, it seems that signaling systems have grown and shrunk, corresponding to the needs of the organism under scrutiny.

## But what is a ‘good’ hormone?

Here, we will discuss a couple of ‘conceptual properties’. The actual chemical properties that might make a good hormone, those determining solubility in water or fat, will only be discussed in the travel section, as they influence where a hormone distributes within the human body and whether that hormone might be able to go through biological membranes or not. The building material for a ‘**good’ hormone** should be readily available or easy to make. A good hormone should have something unique in its structure, enabling its specific recognition unambiguously. It would be handy if a hormone’s action could be modulated in space and time. Probably most importantly, there should be mechanisms in place for switching the signal off again. All these features are important not only for hormones but also for other signaling molecules. Here, we go through all these points again for a primordial hormone, still used as such in some invertebrates – the well-known secondary signaling molecule cyclic adenosine-monophosphate or cAMP. Interestingly, vertebrates have internalized cAMP – in us, it is an intracellular second messenger, a hormone-of-a-second kind. Second messengers other than cAMP will not be covered in this article.

Cyclic AMP can **easily be made** from the omnipresent precursor ATP – the cAMP-producing enzyme adenylyl cyclase will produce cAMP wherever and whenever it is instructed to do so by upstream signaling. Cyclic AMP is quite an interesting chemical compound because next to its well-known five-membered ribose ring, it has the newly formed internal phosphodiester six-membered ring. The two rings cling to each other tightly. In fact, there are not many chemicals that look or behave distinctly similarly; maybe with the only exception of cAMP’s cousin, cyclic GMP, which just contains a different nucleobase. This all means the molecule has a very **specific chemical signature** or ‘fingerprint’. The signaling molecule cAMP exclusively targets two families of responsive proteins, cAMP receptors of some kind. One of these families includes the many different isoforms of protein kinase A. The other one is the cAMP-regulated guanine nucleotide exchange factor, Epac for short, an odd protein that connects two signaling ‘worlds’ – cAMP signaling and the action of small G proteins. In this signaling system, cAMP, the signaling molecule, is **recognized with high specificity** by its cognate receptors, protein kinase A and Epac.

It is handy that this signaling can be tweaked or **modulated in space and time**, adapting a molecular message to the specific biochemical needs of a certain tissue or organ. Protein kinase A itself is already a complex system of regulatory and catalytic subunits. Special tethering proteins, however, make its signal transduction even more intricate – A-kinase-anchoring-proteins. These proteins pull together the kinase and some of its substrates, but not others, allowing for very specific signaling events confined in time and space. Finally, the biologically potent cAMP signal can be **switched off** again with ease. Dedicated phosphodiesterase enzymes can cleave the internal double ester from within cAMP rather quickly, as if nothing ever happened.

Taken together, a hormonal message should be easily synthesized. The molecule should be unique to a certain extent, allowing for specific recognition by specialized receptor molecules. Ideally, the hormonal system should offer ways of modulation in space and time. Definitively, effective ways need to exist to make a hormonal message undone – some sort of degradation pathway. Each of these points might also be interpreted more broadly – so in addition to being easily synthesized, one could also add some sort of chemical stability to that list. Can you discover some of these features in other hormonal signaling systems?

## How does a cell produce a hormone?

Hormonal signaling is largely mediated by ‘**standard’ metabolites**. Peptide hormones and glycoproteins, for example, are easy-to-make in principle – but complicated-to-make in practice. Think of β-cells from within the pancreatic islets of Langerhans. β-cells are massive protein factories for a single thing – **insulin**. So, all the processes in the multistep conversion of the insulin gene, via the splicing of its mRNA, its translation, proteolytic processing, oxidative folding, and packaging into secretion vesicles need to run smoothly and be coordinated. Because this process has been known in much detail for a while, the presence of the C-peptide was used to test for endogenous insulin production for the diagnosis of diabetes. Nevertheless, any disturbance to this machinery, such as endoplasmic reticulum stress, might lead to β-cell senescence and breakdown, leading to diabetes. Part of this stress on the insulin production ‘machinery’ may lead to the generation of neo-antigens, putatively by out-of-frame translation of the insulin mRNA, making the β-cell more immunoalert than an average cell. So, even making a lot of a ‘simple’ thing can turn out to be challenging. All in all, the relationship between β-cells and the immune system has been called a chicken or egg dilemma. Are β-cells weaker than other cells, or are they so exceptionally hardworking to make them immune targets only secondarily? A lot of insulin and diabetes research is about this question.

**Figure 5:**
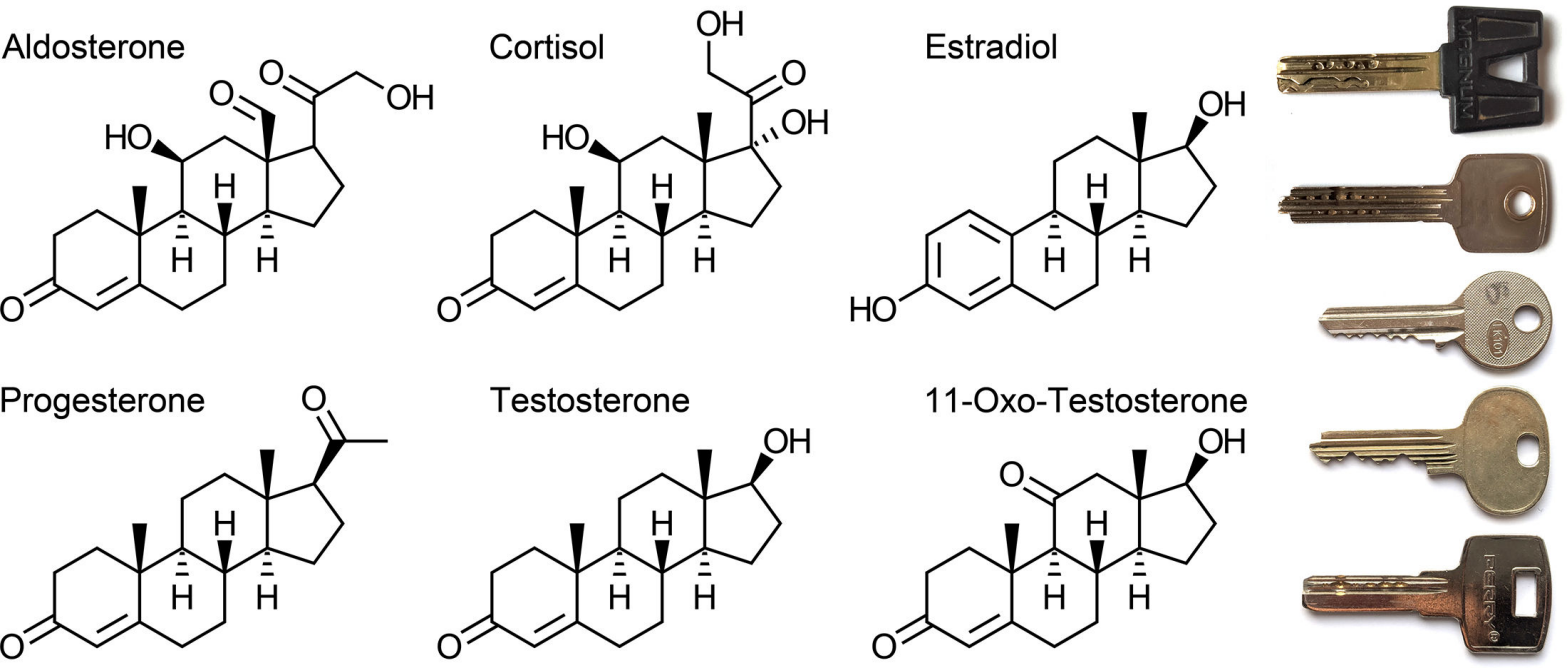
Are steroids “good” hormones? There is a whole group of steroid-based signaling molecules: the mineralocorticoid aldosterone regulates salt balance and blood pressure. The well-known stress hormone cortisol makes us fit for a stressful life. Estradiol, progesterone, and testosterone all are linked to sexual development and reproduction. And we have vitamin D3 – the sunshine hormone, depicted in Figure 3. Instead, we add 11-oxo-testosterone, an androgen discovered more recently. For all of them ‘carved’ out of easily available starting materials, such as cholesterol, one may consider that these hormones can be produced easily, as long as the steroid-producing cell is well protected from the harsh oxidation events here or there. The stable scaffold with not too many variations on some features makes steroids somewhat similar to security keys. Fun fact: Did you know that when arrayed alphabetically, including ‘calcitriol’ for 1,25-vitamin D3, the first letters read ‘A-C-C-E-P-T’? Would this be useful to make a mnemonic out of it? Image credit: Jon Wolf Mueller and Dominic C. Y. Lai

**Steroid hormones** are made from cholesterol, certainly a central metabolite, vital for our body. Most of our daily cholesterol comes from our diet; this can be even too much at times. Our own body can make cholesterol as well, employing pretty fascinating biochemistry, not covered here. It is this body-made cholesterol that cholesterol-lowering drugs are targeted at. The steroid scaffold that cholesterol has to offer, which gives rise to some of the most powerful signaling compounds in the human body, are made – only with “some” modifications – by removing a methyl group here and oxidizing a carbon atom there – some steroid hormones are shown in Figure 5. The relatively ‘stiff’ cholesterol molecule undergoes several biochemical transformations, mainly catalyzed by two classes of enzymes, the hydroxy-steroid-dehydrogenases (HSDs) and the cytochrome P450 enzymes. Biochemically, the HSDs are clean and precise, so to speak. Apparently, these enzymes are even able to catalyze their respective reaction in either direction, depending on whether they use NAD or NADPH as a cofactor. It is this tapping into different pools of redox cofactors that allows HSDs to ostensibly be different from other enzymes; those that catalyze the reaction in both directions and do not affect the equilibrium.

And then, there are the somewhat rough cytochrome P450 enzymes, the cyps, with a tightly bound heme cofactor. Cyps bind and activate molecular oxygen, only to catapult it then at a specific site of their bound substrate, more or less specifically. While doing so, cyps drop quite a few electrons here or there; this is why they are regarded as rough in this article. In addition to those reaction products found in textbooks and depicted in Figure 5, steroid molecules with ‘unusual’ oxidation patterns arise, such as 11-oxo-testosterone. During steroidogenesis, lots of reactive oxygen species are created as by-products, making steroid production normally not compatible with smooth and ‘healthy’ cellular metabolism. This is probably why steroid production is confined to only a few tissues, such as the outer shell of the adrenal gland. How other tissues get the steroid hormones they need will be discussed in the transport chapter.

At the opposite end of ‘standard’ metabolites, probably is the making of the **thyroid hormones**. Starting off maybe in deep ocean waters, organisms enriched with iodine and connected it to amino acids. Some jellyfish organize their asexual division by thyroxine, even though they clearly lack some components of the thyroid hormone signaling system. Many, many steps of evolution later, we end up with our thyroid gland that can enrich iodine from blood, using a specific uptake transporter. The cells within the thyroid form small bubbles, and the inner enclosed space is where thyroxine is made. That space is filled with a humongous large protein giant – a dimer of thyroglobulin with more than 2500 amino acids each and a total molecular weight of about 600 kDa. Tyrosine residues are first decorated with iodine; some textbooks call this process the organification of iodide. These tyrosine side chains are then combined to make protein-bound ‘tyrosyl-tyrosine’, which then just needs to be proteolytically released. There we are – a lot of T4 and a bit of T3 are made. What could be easier? The tyrosyl transfer chemistry is quite harsh, so again, that reaction is confined to the inner part of the follicle; or more precisely to the interior of thyroglobulin itself, it catalyzes the transfer of up to six tyrosine residues to their respective twin tyrosine in a radical reaction mechanism. At the same time, thyroglobulin is the reaction solvent, as all reactions take place within the protein, and additional iodinated tyrosine residues, not involved in T3/T4 synthesis, may function as a storage for iodate. Taken together, making a hormone can range from easy to extremely elaborate.

## And what are endocrine glands good for?

Making certain hormones requires tissue-specific **protection and containment**, as seen above. Such a need for encapsulation may have led to the development of endocrine glands – discrete anatomical entities that are structurally and functionally different from surrounding tissues. Not only aggressive chemistry but those powerful hormones themselves, might be stored in this way. Compared with hormone-producing cells scattered randomly over the body, endocrine glands may come with several other advantages as well.

If a certain cell has the biochemical capacity to synthesize a certain hormone, and its general fitness allows for it, this cell will produce that hormone in **response to an appropriate signal** from the outside, be this hormone good or bad for the body. A big topic in this regard is *endocrine cancers* that mass-produce a certain hormone at times, causing clinically severe conditions of hormonal excess. For this chapter on glands, though, it is the response to an appropriate signal to be discussed. Glands are, however, not only responding to one ‘main’ message but they are also metabolic integrators of a rich language of hormonal messages and metabolites with a built-in feedback function. Being together in a gland, endocrine cells can respond synchronously to external signals.

In addition, being clustered together in a gland allows endocrine cells to communicate with each other directly through chemical signals. A remarkable example of such a **hub for hormone production** is β-cells in the pancreatic islets of Langerhans – they talk to each other to coordinate their insulin secretion; there are even some β-cells that take the lead. This allows for a quick, precise, and coordinated release of hormones based on the body’s needs. Such a setup may lead to all sorts of dynamics during hormone secretion. Traditionally not seen, or leveled out, in spot measurements or 24-hour urine collections, we know of more and more hormones that may be secreted in pulses, and those patterns might transport additional information.

**Figure 6:**
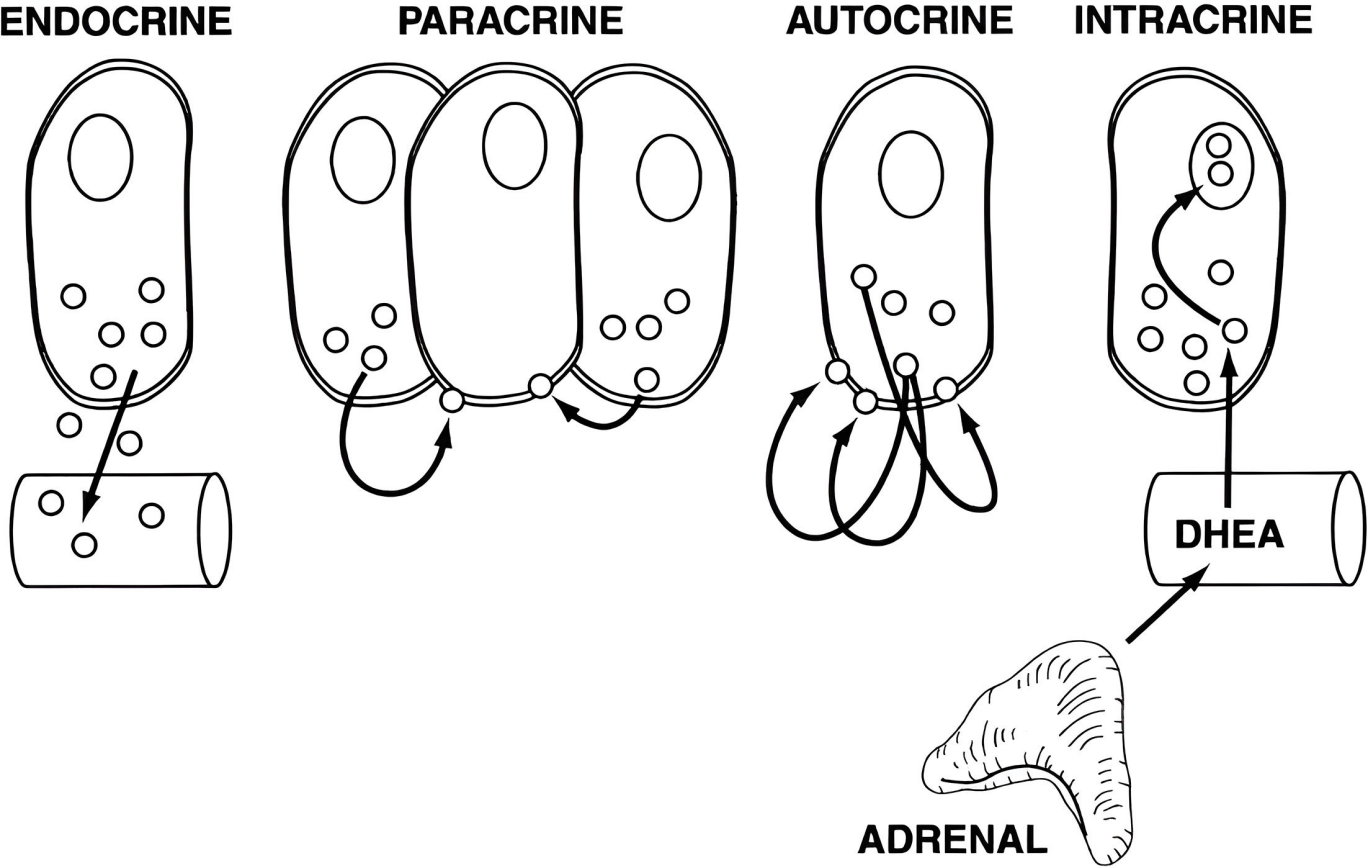
What is an endocrine gland? Chemical compounds are secreted by all sorts of cells. Depending on how far they travel to their target cells, endocrinologists use all sorts of technical terms. These are shown here in a schematic by Ferdinand Labrie, already in 1991, and again in a modified form in 2003 (reprinted with permission by Oxford University Press). Some named him the ‘inventor’ of intracrinology.

Then, there is a **transport and delivery** aspect. Endocrine glands tend to be situated close to a rich network of capillaries, allowing for the quick entry of a secreted hormone into circulation. On the opposite, if a gland is anatomically coupled to some other tissue, the action of that hormone might be directed to some other cells specifically. All in all, distributing hormone production throughout all of our bodies, probably would make hormonal endocrinology weaker and slower-acting, more difficult to regulate, and might have damaging consequences for the actual endocrine cells due to some complicated biochemistry happening. It is the section on glands, we have attached a schematic that differentiates endocrine actions of signaling compounds from other modes (Figure 6).

## A tale of axes, cascades, and avalanches – secretion of hormones, signal amplification, and negative feedback

If there is a demand for a hormone, the responsible cell should release the said hormone. Sounds easy? It certainly is a lot, but easy. Most often, the cell that perceives the signal for the physiological need is different from the cell that is supposed to secrete the corresponding hormone. And there is a lot of ‘talking’, of signal sending, receiving, and integration, in between. So, broken down to the individual cell, or the cellular context of a gland, it is normally an upper-level hormone that gives the thumbs-up to blow out the hormonal message. This **hierarchy of hormones** is what prevails in first- and second-year classes of molecular endocrinology.

For the fight-or-flight response, a burst of cortisol is needed. We mentioned cortisol already – a wonderful example of massive **signal amplification**. It all starts somewhere in your brain, receiving sensory input of imminent danger. This signal then is sent to the hypothalamus, a pea-sized extension of some other brain structures. In response, the hypothalamus releases a peptide called corticotropin-releasing factor or CRH, in tiniest amounts, often labeled as ‘not detectable in serum’. This CRH-releasing factor directly stimulates the pituitary, in a targeted way, because the hypothalamus and pituitary are connected directly by a portal vein (discussed in the transport chapter). The pituitary then produces a lot more of another peptide called adreno-cortico-trophic hormone or ACTH. ACTH then is flushed into the general circulation. Some ACTH molecules reach the adrenal cortex and activate the ACTH receptor – ACTH arrives at the adrenal cortex at concentrations between 20 and 100 pico-moles per liter. Immediately, one part of the adrenal gland, the *zona fasciculata*, secretes micromolar quantities of cortisol. On a busy day, ACTH triggers the release of cortisol at blood concentrations between 80 and 700 nanomoles per liter; that is more than 1000-fold more molecules involved. Linking back to Figure 2, where an electrical signal started thas cascade, amplification factors in this system are humongous large.

**Figure 7:**
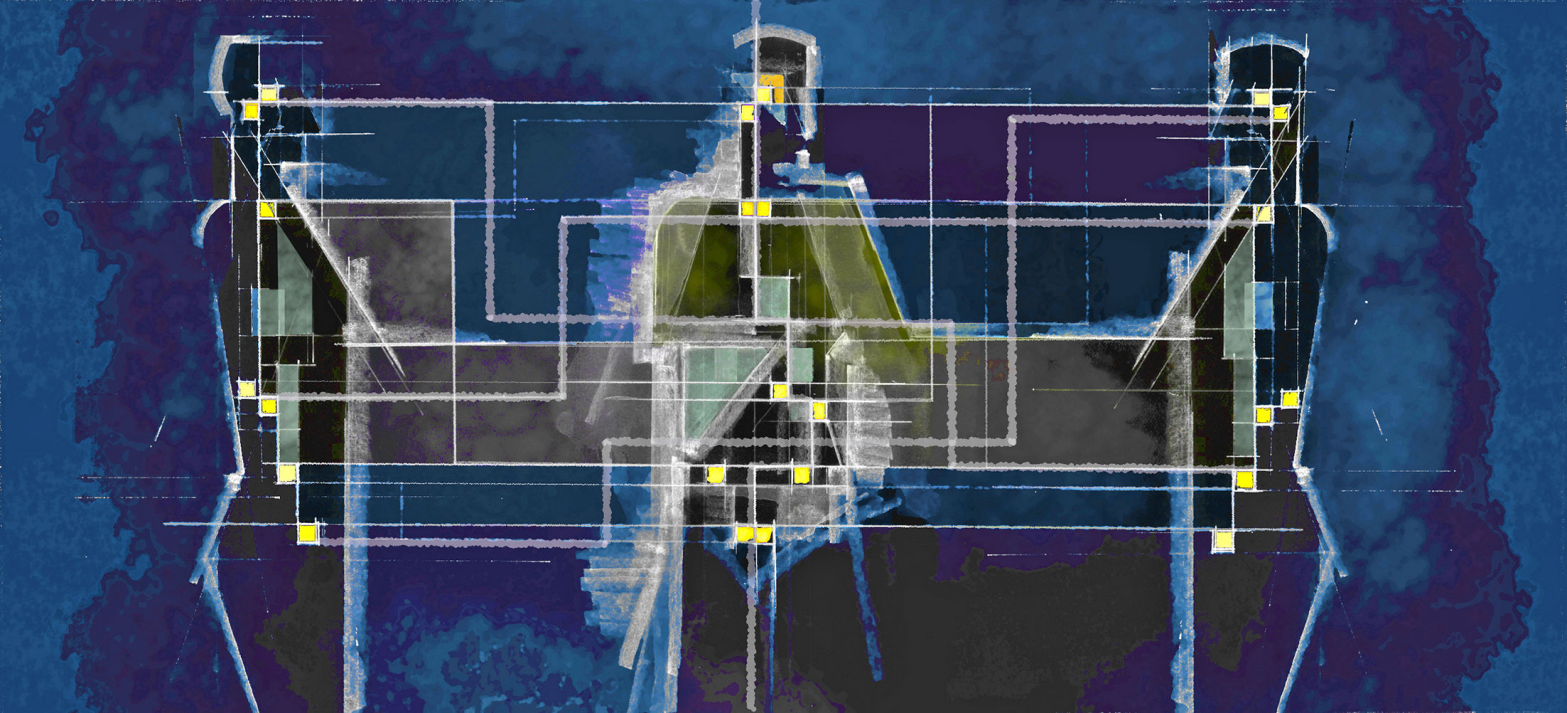
Axes everywhere. An artistic representation of the human endocrine network where glands are connected by molecular tracks. The present article invites endocrine education to explore other metaphors than axes. See main text for details. This artistic representation of the endocrine system, though, shows bright endocrine hot spots of color connected in various ways between androgynous figures. The piece is produced by artist John Gage using a combination of hand drawing, painting, and digital media. It is also a homage of sorts to Piet Mondrian’s Broadway Boogie Woogie (1943), in its representation of the traffic of the human body and a system of jagged interactions reminiscent of jazz music. Image re-used with permission from Bioscientifica

**Axes** are everywhere (Figure 7). It seems unclear who introduced the concept of axes, endocrine axes, into endocrinology. For an endocrinologist, the word ‘axis’ probably is best represented with ‘connectedness’; suggesting that some endocrine gland somehow influences another. For mathematicians and engineers alike, axes are something straight, connecting two dots, or wheels, and maybe allowing rotation around these… Ideal fodder for misunderstanding, as the hypothalamus–pituitary–something-else axes can never be straight lines. No doubt, there are upper- and lower-level endocrine organs, but these certainly are not lower-class organs. The massive amplification described above certainly allows for being fascinated by the adrenal’s biochemical capabilities. Maybe we should move away from calling endocrine systems ‘axes’. **Cascades** of enzymes might be better… Or even the concept of an **avalanche** – a footprint slightly off from your track, or a pebble thrown the wrong way, might launch an avalanche of snow.

It is the amplification power of these systems that calls for surveillance – **negative feedback** loops are perfectly necessary for healthy hormonal signaling. To a good extent, such feedback is easy to establish. As an example, let us think of the upstream hormone to be a peptide and the downstream hormone to be derived from amino acids – we are at the thyroid-stimulating hormone (TSH) and the thyroid hormones. The whole feedback loop only consists of TSH-producing cells, that respond to T_3_/T_4_; these cells will then activate nuclear receptors for thyroid hormones, and these receptors will regulate the TSH-coding gene itself, among many, many other genes. In principle, this is quite a simple regulation loop. In practice, the system is more elaborate because of other factors such as deiodinating enzymes. We have arrived at individualized medicine, where each of us, at a certain age, has an individual reference range for some hormonal signaling compounds.

A final aspect of higher-level hormones is that these hormones tend to convey two different kinds of information. As seen above, endocrine cells like those from the adrenal gland, wait for a specific signal to become active. This signal can be called a **tropic hormone** – a hormone coming from somewhere else, targeted at the specific gland, here the adrenal gland. The proper tropic signal for those adrenal cells from a higher-hierarchy endocrine cell is ACTH. ACTH, in addition, acts like a growth signal for the adrenal gland; ‘asking’ the adrenal something like *Dear gland, we need you, please GROW and stay healthy*. ACTH in this context is a **trophic hormone** for the adrenal.

Textbook examples of **tropic** hormones are TSH, ACTH, LH, and FSH.

Textbook examples of **trophic** hormones are TSH, ACTH, LH, and FSH.

Have you noted the difference? Is it worth memorizing these two terms? Probably not. Is it important in endocrinology that most higher-level enzymes stimulate hormone secretion AND the growth of the gland they are targeting? Much more likely so!

## How does a hormonal message travel? How to make a hormone fit to go with the flow…

Thinking of our message-in-a-bottle, it cannot choose where to travel to. It travels the seas completely directionless. There are, however, features of the coastlines, currents, or even channels creating currents, all to create some sort of directedness to the hormonal message.

NO hormone ‘goes’ straight to where it is most ‘needed’. For any compound freely floating through the human body, rules of pharmacokinetics apply. These principles cause certain compounds to be distributed to some bodily compartments and other components to reach different parts of the body. Again, any hormonal compound passively diffuses through our body. Some hormones can do so on their own; they are reasonably soluble in serum and hence will be brought into the tiniest of our many capillaries. Let us think here of insulin again or maybe adrenaline and noradrenaline. Whether soluble in water or fat, again, is not a choice, but dictated by the chemical composition of the hormonal signal. As an exercise, maybe highlight HO-hydroxyl groups and less polar C=O keto groups in the different steroid structures provided in Figure 5. Counting them, could you guess the love for fat, ‘lipophilicity’, of these compounds? **Differences in solubility** have a massive consequence for these compounds. The steroid that passes freely through biological membranes is a concept that needs to be retired. Maybe applicable to a fraction of steroid compounds only; however, most need dedicated transporters to cross through membranes. Even regardless of biological membranes, differences in solubility can dramatically change a molecule’s path through the human body. A water-soluble compound may only be able to distribute among the 5-or-so liters of blood, while a fat-loving compound may diffuse into all membranes, all our fat tissue, and the skin.

Within our circulation again, some other hormones need the help of **transport proteins** to become reasonably soluble in serum. The prime transport protein in our blood is albumin, a protein forming some sort of a sponge, with a number of hydrophobic pockets. Nutrients and hormones alike can bind to albumin, enjoy being transported in the circulation, and then dissociate somewhere away from where they initially encountered albumin. Please bear in mind that albumin does not confer any direction to the diffusion; it simply extends a molecule’s half-life in serum.

More specifically, some of the **transport proteins** that can carry certain substances might be confined to certain parts of the human body. Carrier proteins for a very bespoke cargo might be hemoglobin and myoglobin, bringing oxygen from the lung to muscle tissue. Conceptually related, we have three different protein carriers for the hormones from our thyroid: the first one is the above-mentioned albumin. Then, there is thyroxine-binding globulin in serum, and transthyretin (also known as pre-albumin). That last one, transthyretin, probably is the most unusual of the three carriers – enriched in the cerebrospinal fluid; this protein binds thyroxine T_4_ very tightly (with high affinity); it may facilitate T_4_’s uptake into the brain.

In our body, there are diffusion facilitators ‘installed’ at some points. Similar to the hepatic portal vein that links all unpaired organs in our belly to liver, there is another portal vein that links the hypothalamus and pituitary. A **portal vein** can be understood as a blood vessel that carries blood from one tissue with capillaries, not directly to the heart, but to another capillary bed… Functionally, the adrenal cortex and the adrenal medulla are linked as well – this, however, is not regarded as a portal vein. Nevertheless, hormones from the hypothalamus are seen first, and nearly undiluted, by the pituitary; similarly, cortisol from the adrenal cortex, first and foremost, makes the adrenal medulla secrete adrenaline. These diffusion shortcuts make some of the bodily fluids flow in some directions but not in others. Similar to the transfer of a molecule from one to another carrier protein, which may appear as a directed process, the overall distribution of compounds remains directionless.

Maybe the most taught shortcut between endocrine glands, hormonal centers, is the connection between the hypothalamus and the pituitary. They are connected by the **hypophyseal portal system**. This means, there are capillaries within the hypothalamus that centralize to a vein, only to form another capillary network at the pituitary. This also means that any secreted molecule from the hypothalamus directly reaches the pituitary, without dilution in the central circulation. Practically, this also means that the hypothalamus secretes releasing hormones, such as those targeting the adrenal and the thyroid, CRH, and TRH, respectively, in such minute quantities that they tend not to be detectable in blood.

Maybe a lesser-known **link between the two hormonal systems** sits in the adrenal gland. Its capillary network is directed from the cortex to the medulla, where the adrenal vein starts. These capillaries connect the cortisol-producing zona fasciculata with the medulla, which is a specialist in producing adrenaline and noradrenaline. Because the capillaries do not merge into a vein in between, this system is not known as a portal vein. Still, this architecture brings adrenaline production under the direct and undiluted influence of our body’s cortisol secretion. Any shock, exam, or fight-or-flight trigger for a cortisol spike directly brings us also an adrenaline rush.

The opposite of any shortcuts in diffusion might be **blocks to diffusion** – parts of the human body where standard metabolites may have a ‘rough’ time getting in. This as well is the opposite of confinements in endocrine glands; again, it now is about controlled entry, not controlled dispatch of hormones. The two most notable blockages may be the blood–brain barrier and the placenta, separating off from normal circulation our central nervous system and the growing fetus, respectively. The benefit of these barriers appears obvious; not everything that normally circulates should interfere with brain function or with reproduction. There are more diffusion barriers, and there are all sorts of additional cells coating blood vessels, special cell–cell attachment points, and dedicated transport systems. Let us leave it there for now.

This section opened with counting HO and C=O groups. We close by discussing if there are charged amino or carboxy groups in our signaling compounds. This paragraph is again about solubility, but with added complexity, and… It is a trap, an **ionic trap**. Whenever low-molecular weight signaling compounds can toggle between a charged state and an uncharged one, that compound will accumulate in the bodily compartment where its charged state – the ion – is favored. If your molecule contains amino or carboxy groups, these might be able to take up a proton or lose one. Be it a positive or negative charge to gain, that electric charge would dramatically increase the molecule’s solubility in water, reducing its fat solubility. Proton-giving or -losing groups of various hormone molecules work at different pH values. A striking example in this regard is the thyroid hormones T_4_ and T_3_. While T_3_’s phenolic hydroxyl group shows a ‘normal’ or expected pK_A_ value of 8.4, well in the basic range, T_4_ surprises us here with a p*K*_a_ value of 6.7. This means that T4, secreted from the thyroid gland – perfectly identical with the widely used drug levothyroxine – is largely negatively charged at pH 7.4, the physiological pH value. Taken together, ionic trapping can confer an apparent directionality of diffusion processes toward some bodily compartments, but not others. Can you imagine what this ionic trapping alone might mean for the signaling properties of T_4_ and T_3_? Enough for now about sending our hormonal message; where is the receiving end, the receptors?

## Where are the receptors?

**Figure 8:**
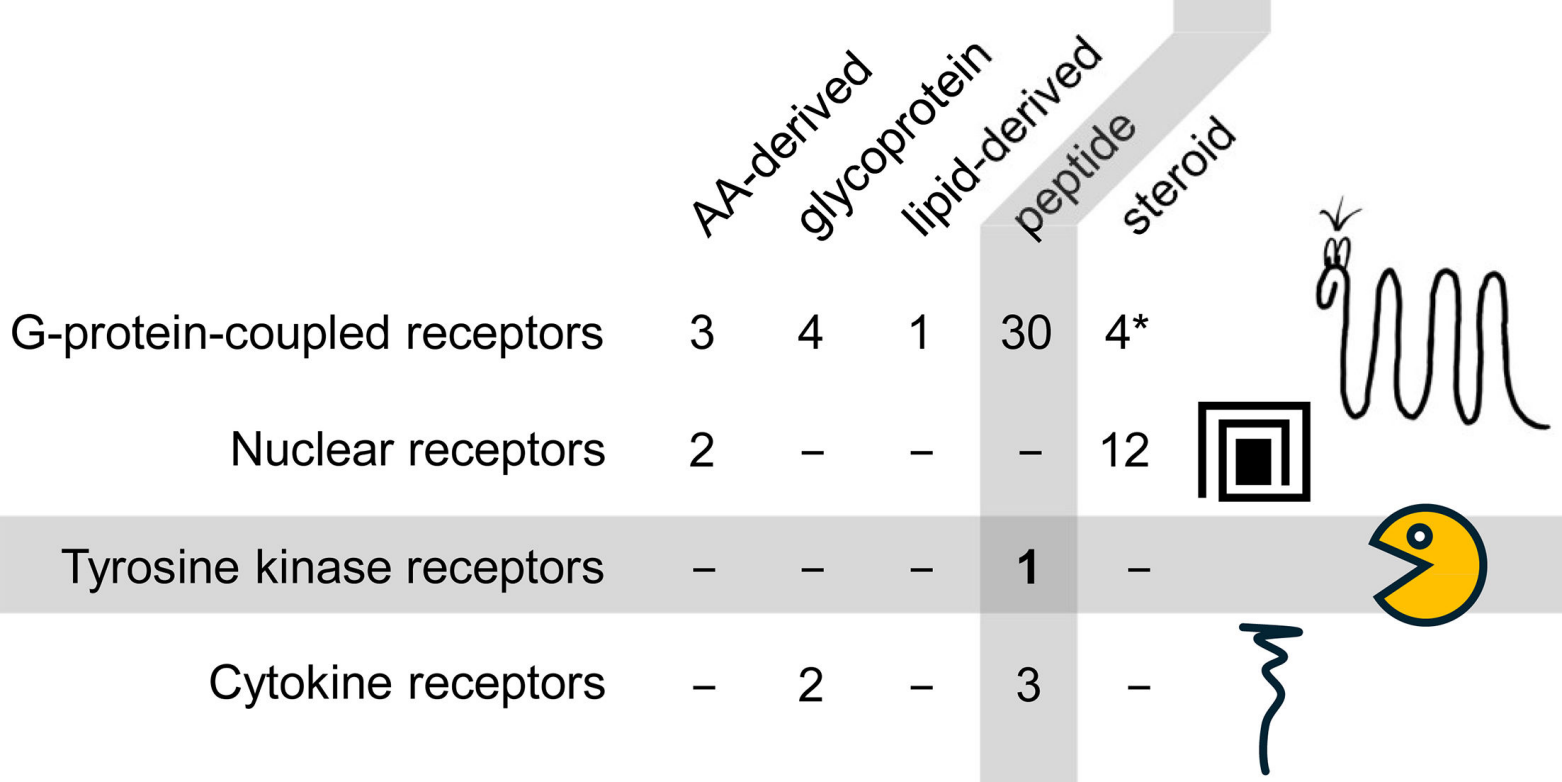
Only four different classes of hormone receptors exist in our bodies. The hormones from our initial list are grouped into five groups according to their chemical makeup. For each of the hormones, the main receptor class is shown. The figure should mainly indicate that the classification of receptors is highly beneficial to learning. The receptors along with some sorts of iconic representations are described in more detail in the main text. A nuclear receptor’s ligand binding domain is depicted as a line wrapping around a central square. The ubiquitous topology of a receptor to be coupled to G-proteins is that it has seven membrane-spanning α-helical segments, not more, not less. Enzymes that double as receptors tend to be receptor-tyrosine-kinases. Can you guess which hormone it is that exclusively couples to such a kinase receptor? And then, there are cytokine receptors, long, somewhat boring peptides that provide docking sites for a lot of signaling later on. **Disclaimer:** There certainly are more receptors in the human body, e.g. when sensing damage or in the field of immunology. And the hormone-receptor pairing might be more complex than shown here. Estradiol, for example, not only interacts with the estrogen receptor, a nuclear receptor, but also with GPER, a G-protein coupled receptor. Four GPER ligands are indicated in the matrix.

So, we need to talk about receptors. For pharmacologists, the above question can be overwhelming to describe what a biological receptor is – it is just any structure a drug needs to bind to, to cause its pharmacological effect. For endocrinologists, especially when just looking at our list from the start, the world is somewhat simpler. For any hormone from that list we started with, the **receptor class** is depicted in Figure 8. There are not many receptor classes. Hence, the simple answer to the old question of why we have to classify receptors at all: it is worth repeating receptor classification thoroughly – you will hear one of these keywords and you will be able to predict quite some biological and mechanistic characteristics. This section will briefly introduce these four classes. We will close with some thoughts on connections between hormonal systems and how specificity might be generated.

Figure 8 suggests that some receptor classes are used more often than others. Even though biology cannot be captured quantitatively in this regard, we start with **G-protein-coupled receptors**, GPCRs, as they are much talked about. These transmembrane receptors most often have a large N-terminal ligand-binding business end. In the middle, they have seven transmembrane spanning helices, less serpent-like, but these seven helices always form a stable bundle of a central helix, with six helices packed around it, as several 3D structures of some of these receptors have shown. And then, there is the C-terminal domain that couples to switch-like trimeric G-proteins – with a GTP nucleotide they are ‘on’, with a GDP nucleotide they are ‘off’. The C-terminal part of the receptor makes the G-protein toggle between the two states, thus transducing its own activation state. A modular toolbox of adaptor molecules translates the signal into a cascade of second messengers. G-protein coupled receptors are a highly abundant class of receptors, also outside of hormonal signaling.

Next on our list are **nuclear receptors**. Their name makes them different from all other classes of receptors, they do not sit in the membrane but act in the cellular nucleus. There are 48 genes for nuclear receptors in the human genome. An example of this class of receptors is the vitamin D receptor. Nuclear receptors consist of several building blocks: one of them is a DNA-binding domain, and another one is the ligand-binding domain.

Confusingly, nuclear receptors do not need to reside in the nucleus to be nuclear receptors. Instead, the ligand binding domain might be unfolded, and such a wobbly peptide chain is guarded by folding-helper proteins, all within the cytoplasm. Other nuclear receptors are kept inactive in the nucleus, complexed to corepressor proteins. When the natural ligand of a nuclear receptor comes, the ligand-binding domain folds around that compound – depicted as that line wrapping around a central square. Now, a properly folded protein, the nuclear factor, is released from the folding-helper machinery, and it migrates to the nucleus. Nuclear factors may form dimers or oligomers with themselves, or even with other nuclear receptors, thus increasing signaling diversity. The last thing to say is that nuclear receptors are ligand-switched transcription factors – no additional second messengers are needed. They themselves influence the transcription of hundreds or thousands of genes everywhere in the human genome.

Then, we have **cytokine receptors**, somewhat boring peptides at first glance, spanning the membrane, not able to do much. The receptor for erythropoietin is an example of a cytokine receptor. If a ligand binds to the extracellular site, two receptor molecules come together, and this brings associated enzymes, carried along on the intracellular part, into close proximity. These enzymes then activate each other; they are kinases, and in some sort of self-referential way, they are switched on by phosphorylation. Then, these kinases phosphorylate a good handful of other sites within the receptor where more signaling molecules bind. The receptor senses the ligand, transduces the message into the cytoplasm, translating it into receptor dimerisation. Then, it provides docking sites for a lot of proteins, for downstream signaling events. Not that boring at all. What do you think?

Last on our list are **enzymes that are receptors**. The single example from this category among our list of hormones is the insulin receptor. It is a receptor tyrosine kinase. Somehow similar to cytokine receptors, with the difference that the domain with enzymatic activity is part of the receptor itself, part of the same polypeptide chain. Otherwise, similar to the above, the ligand – it is insulin here – binds to the extracellular domain; somehow this event is signaled to the inside, where a lot of phosphorylation occurs, triggering off some other signal transduction.

For the action of all our hormones, we only need to consider four classes of receptors. Should we extend into immunology, stem cells, or pharmacology, one needs to deal with **more kinds of receptors**, with quirky stuff. Things not covered here are binding kinetics and affinities between hormones and their ligands.

There is the ever-recurring question around specificity – one story in the next section will focus on this. An article in its own right could be about the linkage between hormonal systems, such as the impact of high cortisol on the thyroid gland. This section ends with signal-modulating extracellular proteoglycans. It does matter if a cell has a lot of carbohydrates around it, or not, because that glycan hull may influence hormonal signaling indirectly – a lot of it might make a signaling paracrine, just limited to some neighboring cells; less of it might make the same signal enter circulation for systemic signaling. One might call these glycosaminoglycans co-receptors of some sort.

## And finally, how to switch off hormonal messages…

Thinking of our message-in-a-bottle, if it is found only many, many years after it was posted into the ocean, the content of the message may have become meaningless.

Any message has a shelf-life… Each hormone, every signaling compound, has to be made undone in some way or another. Surprisingly, the **kidney** is a powerful organ regulating retention rates in our blood. The kidney’s glomeruli ruthlessly **filter** out almost everything up to a molecular weight of 30–50 kDa into the primary urine – all goes away, useful drugs and toxic compounds, all at the same time. But then, a plethora of transporters recycles ‘useful’ things back into our body. This two-staged urine production is very good in eliminating nearly everything that the body ‘does not know’ molecularly, swimming in our bloodstream. Even the fanciest designed drug-like molecule will be secreted this way unless it has some molecular similarity to some of our standard metabolites. Then, that drug would be reabsorbed alongside that metabolite. Excretion via bile and other bodily fluids play its role here as well. The one thing that does not happen in our body is a targeted destruction or excretion of ‘unwanted’ molecules.

**Liver**-allocated **biotransformation** processes are a more biochemical option for switching hormonal signals off. The liver stocks a good panel of biotransformation enzymes, all only too ready to spring into action once ‘stuff’ comes along, be it from blood in general, or more specifically from the portal vein, which directly connects all unpaired organs in our belly with the liver. Most of these enzymes can oxidize compounds or link them up with charged metabolites. These two reactions have been called ‘phase I’ and ‘phase II’ in the past. They, however, happen at the very same time, and they have the same effect on small molecules – the liver transforms these into more charged, more water-soluble downstream products; most likely now better suited to be secreted by the kidney. Those oxidizing and conjugating enzymes tend to be notoriously promiscuous enzymes, at least to some extent. They will act on anything that fits their substrate profile, be it good or bad for our body. As we understand conjugation processes in more and more molecular detail, it becomes possible to design hormonal medications that rely on activation by liver enzymes, so-called pro-drugs. The opposite has also been described – to extend a drug’s half-life within the body by making it escape certain kinds of liver biotransformation.

Now moving to the rich world of transmembrane receptors. What do our cells do when a hormone has engaged with a receptor? The answer depends on how tightly the binding might be. Transient and low-affinity binding leave the receptor ready for another binding event. More sticky binders cause something rather strange – receptor activation not only starts the respective signaling, it also leads to the recruitment of recycling proteins, such as clathrin. The cell then absorbs the **receptor–ligand complex** as a membrane-wrapped vesicle, an early endosome, that soon will be **degraded**. Receptors for insulin, glucagon, and adrenaline are examples of such ‘signaling with appetite’.

The steroid hormones contain a fantastically bad design in hormonal specificity that requires **targeted degradation of a hormone** for the system to work properly. It happens that our mineralocorticoid aldosterone is not that different from our glucocorticoid cortisol. In fact, these hormonal systems have diversified only recently within the mammalian lineages. This means that large quantities of cortisol actually can activate the mineralocorticoid receptor. However, there is a biochemical trick in the kidneys; that is where blood pressure and salt homeostasis are regulated by the mineralocorticoid receptor. These tissues express quite some levels of a specific type of the enzyme 11β-hydroxy-steroid-dehydrogenase – 11β-HSD type 2. This enzyme disarms cortisol by converting it to inactive cortisone. Thus, the mineralocorticoid receptor is protected from cortisol and can do its job in aldosterone signaling.

## How to study hormone signaling

A lot of hormones have been known for decades. Several steroid hormones, among them estrogens, testosterone, and progesterone, were discovered in the 1920s and 1930s, and cortisol, very soon after its discovery, was celebrated as a wonder drug. Nowadays, many patients are **prescribed glucocorticoids** for various reasons. Synthetic glucocorticoids, such as prednisone or dexamethasone, seem to be nearly as old as cortisol itself. Hopefully, the future will bring powerful cortisol mimics that lack the side effects of the actual compound, those unwanted side effects inducing Cushing-like symptoms.

As we cannot be sure that we know all compounds by now that act as signaling molecules in our body, how have discoveries been made in endocrinology? And how to make them in the future? In the past of endocrinology, there was a strong element of **from bedside to bench**. This means that most observant clinicians spotted the rare patient(s) with symptoms somewhat different from what was in the textbook. Together with expert basic researchers, these cases were then phenotypically and mechanistically investigated. In this way, whole alternative pathways were worked out.

Spotting the rare patient has become an entirely different discipline these days, as **whole-genome sequencing** of a patient (and their relatives) has been put earlier and earlier in diagnostic pipelines. New DNA sequencing approaches have reduced the costs of sequencing a whole human genome to under £500, with turnaround times of less than a day. So, more often than ever before, the researcher needs to make meaningful connections between sequenced genotypes and clinical presentation, sometimes called geno-2-pheno correlations. Humbly, we had to learn that the same genotypes may have drastically different phenotypes, and for genetic diseases with a combination of phenotypes, patients presenting with all diagnostic criteria were the exception, not the rule.

Medical research is not about DNA only; it also is about a lot of analytics, measuring a multitude of metabolites with analytics ranging from traditional immune-sorbent assays to state-of-the-art nuclear magnetic resonance and chromatography-coupled mass spectrometry. **Innovation in analytics** tends to drive discovery here, as in:

*I can only research what I can see (or measure*).

So, once we know in perfect detail what there is in a given bodily fluid, we then can ask about signaling properties of the ingredients just detected. Somewhat related are enabling technologies that might allow hormones to be hidden in molecular cages and then to release them, for example, by a flash of bright light – opto-endocrinology – which might allow very exciting discoveries in the near future.

Clever analytics might leave us with some candidate compounds that we need to test in whatever model system. Traditionally, model systems were those organisms traceable with classic genetics – mice, flies, worms, and yeasts, maybe also bacteria and plants. Choosing your **experimental system** was more dictated by preference and available technology than by the research question. A lot has changed here in the last decade, as new genetic techniques, mostly related to CRISPR-Cas9, make a lot more organisms amenable to gene disruption approaches.

A lot of biomedical researchers also hope to bring discoveries **from the bench to the bedside**. Assuming you have any given system of relevance to endocrinology, and you understand it to a great extent, the chance is that you can use this system for innovation. Improve the receptor. Make a soluble form of the receptor to create an antagonist. Improve the hormone, or at least derive reliable biomarkers to monitor a condition or a disease. Findings need to be published; intellectual property might need protection. Validation studies need to be conducted, and – towards clinical implementation – clinical studies might need to be conducted. A lot of skills are required here, skills that exceed a basic scientist’s and clinician’s skill set, so teamwork is key.

## Conclusion and outlook

Numerous metabolites only wait to be discovered as secondary signals for this or that process. Recent research outputs in this regard have revealed that the following metabolites have **a second function in metabolic signaling**: 6-phospho-glucose, lactate, 2-oxo-glutarate, and succinate from the fraction of small molecules. As a protein signaling factor, the sulfotransferase SULT4A1 stands out. This highly conserved protein, related to sulfation pathway enzymes, was known to have no detectable catalytic activity for quite some time, a suspected secreted factor of some kind. The future most likely will reveal more and more parts of central metabolism to be involved in some sort of signaling, in addition to their known functioning.

While we extend the list of compounds with signaling properties, our imagination of what may constitute a hormone may be vastly challenged, so how about gases? Among the signaling molecules from plants and bacteria, there are several **volatile gases** acting as hormones. The ripening hormone ethylene might be the best-known member from this category; but nitric monoxide (NO) and hydrogen sulfide (H_2_S) are transmitting molecular messages as well… And there is a large number of other volatile signaling compounds linking different players in diverse ecosystems that we only begin to understand. With regard to the gases NO and H_2_S, comparative endocrinology may serve as a generator of research questions. In fact, these molecules play important roles also in human physiology and pathophysiology. One might even ask why they have not featured in the list that we started off with. Most likely it is their short half-life that makes them be classified instead as endocrine compounds, as paracrine signals that travel only from one cell to its neighbor or so.

Quite an interesting topic for future endocrine research might be **human pheromones**. For some insects, we know down to atomic detail what attracts them to each other; knowledge is noteworthily sparse when it comes to human chemicals that influence our bonding in (real) social networks, for partnership, and for reproduction.

Looking at that initial list of human hormones or hormone families and beyond, we can paraphrase Peter Medawar’s quote probably like this: “A hormone is any substance when put into use as a hormone.” May be interpreted as: As long as life evolves as a whole, hormonal signaling systems will continue to evolve to cater ever-changing physiological and environmental demands. Similar signaling compounds may have found different uses in different forms of life. At the same time, different compounds might be used for the same signaling events. Let us hope for a bright future then of **comparative endocrine research** approaches.

**Summary**

- We discuss “Hormone Biochemistry”, coming from new and creative angles, using metaphors, analogies, and elements of storytelling.
- A good part of the article is devoted to the evolution and co-evolution of hormonal compounds and their receptor systems, what sometimes is called comparative endocrinology.
- We go through biosynthesis of hormones, their regulated release from secreting cells, and how hormones travel through the human body, how they activate receptors, and ultimately get degraded.
- A number of hormones are highlighted, such as the family of steroid hormones, with their intricacies around their synthesis, modification, secretion, transport, and signaling.
- We end with an analytical and research methods section around studying hormones, combined with an outlook of what to expect from this exciting research field in the future.

## Further Reading

This is not a classical reference list in the sense of backing up each and every thing written above. Instead, this isan annotated readng list of somewhat thought-provoking articles, loosely following the sections of the articlepresented above.

### Evolution of hormones and hormonal signaling systems

- Mindboggling insights about insulin and insulin-like peptides from worms. They have many more insulin-likepeptides than us: Zhu and Chin-Sang. 2024. C. elegans insulin-like peptides. *Mol Cell Endocrinol.* 585:112173.Review. doi: 10.1016/j.mce.2024.112173.
- A thorough analysis of steroidal enzyme evolution: Markov et al. 2009. Independent elaboration of steroidhormone signaling pathways in metazoans. *PNAS*. 106(29):11913-8. doi: 10.1073/pnas.0812138106.
- A fantastic review on the evolution of the vitamin D signaling system: Hanel & Carlberg. 2020. VitaminD and evolution: Pharmacologic implications. *Biochem Pharmacol*. 173:113595. Review. doi: 10.1016/j.bcp.2019.07.024.
- A thought-provoking journey to the roots of thyroid hormone signaling: Holzer et al. 2017. Evolution ofligands, receptors and metabolizing enzymes of thyroid signaling. *Mol Cell Endocrinol*. 459:5-13. Review. doi:10.1016/j.mce.2017.03.021.
- Malcom Peaker writes about Medawar’s famous quote, as it also had been ascribed to other scientists. Peakerconcludes that the currently available evidence indicates that Medawar’s famous dictum was Medawar’sand his alone: Peaker M. 2016. Medawar’s Dictum on Endocrine Evolution: A Case of Mistaken Identity?*The Endocrinologist*. 121:34. https://www.endocrinology.org/endocrinologist/121-autumn16/features/medawars-dictum-on-endocrine-evolution-a-case-of-mistaken-identity/.

### Glands and concerted signaling

- Pancreatic beta-cells talk to each other, a lot: Peercy & Hodson. 2024. Synchronizing beta cells in thepancreas. *Elife*. 13:e95103. Editorial. doi: 10.7554/eLife.95103.
- Can we measure hormone secretion in timely resolutions, never been able before. Yes: Upton et al. 2023.High-resolution daily profiles of tissue adrenal steroids by portable automated collection. *Sci Transl Med*.15(701):eadg8464. doi: 10.1126/scitranslmed.adg8464.
- Some first insights into the “beta-cell conundrum”. Beta-cells seemingly self-inflict autoimmune attack, byproducing neo-antigens from slippery protein translation: Russ & Davidson. 2021. Found in Translation:Novel Insights into Type 1 Diabetes and beta-Cell Biology. *Diabetes*. 70(10):2185-2186. Comment. doi:10.2337/dbi21-0031.
- And a more recent publication now on the beta-cell “Chicken-and-Egg-Question”: Hansen et al. 2024. TheChicken or the Egg Dilemma: Understanding the Interplay between the Immune System and the beta Cell inType 1 Diabetes. *Cold Spring Harb Perspect Med*. a041591. Review. doi: 10.1101/cshperspect.a041591.

### Traveling of hormonal messages – and the delicate topic of crossing membranes

- A detailed biophysical view on what steroids might do within a membrane: Crowley et al. 2021. Theinteraction of steroids with phospholipid bilayers and membranes. *Biophys Rev*. 14(1):163-179. doi: 10.1007/s12551-021-00918-2.
- A comment on an insect transport protein for steroids: Schweizer et al. 2019. The Ins and Outs of SteroidHormone Transport Across the Plasma Membrane: Insight From an Insect. *Endocrinology*. 160(2):339-340.doi: 10.1210/en.2018-01034.
- They are not all the same, when it comes to crossing cellular membranes: McManus et al. 2019. Rapid andstructure-specific cellular uptake of selected steroids. *PLoS One*. 14(10):e0224081. doi: 10.1371/journal.pone.0224081.
- A quantitative description of the meandering of hormonal compounds within our body: Holt et al. 2019.Methods to Predict Volume of Distribution. *Curr Pharmacol Rep*. 25(5):391-399. doi: 10.1007/s40495-019-00186-5.

### Fine-tuning of hormonal action

- Knowing conjugation on a molecular level allows to design drugs that escape such detoxification processes,thus drastically extending plasma half-life: Cook et al. 2021. Small-molecule control of neurotransmittersulfonation. *J Biol Chem.* 296:100094. doi: 10.1074/jbc.RA120.015177.
- Ursula Klingmüller showed a fascinating new aspect of receptor endocytosis, in 2010. Studying the receptorfor erythropoietin as an example, they showed that receptor internalization leads to completely differentbinding isotherms: Becker et al. 2010. Covering a broad dynamic range: information processing at the erythropoietin receptor. *Science*. 328(5984):1404-8. doi: 10.1126/science.1184913.
- Signaling within the pancreatic beta-cell might be fine-tuned by sulfation pathways: Mueller et al. 2024.Sulfation pathways in the maintenance of functional beta-cell mass and implications for diabetes. *EssaysBiochem*. EBC20240034. Review. doi: 10.1042/EBC20240034.

### A bright future for hormonal research

- Claudio Mauro is a fan of lactate: Certo et al. 2022. Understanding lactate sensing and signaling. Trends *Endocrinol Metab*. 33(10):722-735. Review. doi: 10.1016/j.tem.2022.07.004.
- Ed Chouchani is fond of succinate: Murphy & Chouchani. 2022. Why succinate? Physiological regulation by amitochondrial coenzyme Q sentinel. *Nat Chem Biol*. 18(5):461–469. Review. doi: 10.1038/s41589-022-01004-8.
- A very recent review on the seemingly inactive sulfotransferase SULT4A1: van Waardenburg & Falany. 2024.Sulfotransferase 4A1 Coding Sequence and Protein Structure Are Highly Conserved in Vertebrates. *Genes(Basel)*. 15(7):914. Review. doi: 10.3390/genes15070914.
- The literature on the gases NO and H2S is vast. Here only one review is listed on H2S and its link to bodilyfitness: Wilkie et al. 2021. Hydrogen sulfide in ageing, longevity and disease. *Biochem J*. 478(19):3485-3504.Review. doi: 10.1042/BCJ20210517.
- A thought-provoking read about human pheromones: Wyatt TD. 2015. The search for human pheromones:the lost decades and the necessity of returning to first principles. *Proc Biol Sci*. 282(1804):20142994. Review.doi: 10.1098/rspb.2014.2994.

### Literature referenced within figures

- A prominent figure in the field, at times called “the inventor of intracrinology”, describes their viewsgraphically. Published here: Labrie F. 1991. Intracrinology. *Mol Cell Endocrinol*. 78(3):C113-8. Review. doi:10.1016/0303-7207(91)90116-a.
- And again here, slightly modified: Labrie et al. 2003. Endocrine and intracrine sources ofandrogens in women: inhibition of breast cancer and other roles of androgens and their precursordehydroepiandrosterone. *Endocr Rev*. 24(2):152-82. Review. doi: 10.1210/er.2001-0031.
- For a stimulating and productive Science & Art collaboration around sulfation of hormones, please readmore in this editorial to a special issue: Mueller & Foster. 2018. Steroid sulfation research has come a longway. *J Mol Endocrinol*. 61(2):E5-E6. Editorial. doi: 10.1530/JME-18-0109.
- You can find more about this and related projects here: Mueller & Gage. 2024. Walking along sulfationpathways – a personal journey. *Biochem* (Lond) 46(3):11–16. doi: 10.1042/bio_2024_144.

## Abbreviations

ACTH: adreno-cortico-trophic hormone
CRH: corticotropin-releasing factor
GPCR: G-protein-coupled receptors
H_2_S: hydrogen sulfide
HSD: hydroxy-steroid-dehydrogenase
NO: nitric monoxide
TSH: thyroid stimulating hormone

